# Evaluating single molecule detection methods for microarrays with high dynamic range for quantitative single cell analysis

**DOI:** 10.1038/s41598-017-18303-z

**Published:** 2017-12-20

**Authors:** Ali Salehi-Reyhani

**Affiliations:** Department Chemistry, Institute of Chemical Biology, Imperial College London, London, SW7 2AZ UK

## Abstract

Single molecule microarrays have been used in quantitative proteomics, in particular, single cell analysis requiring high sensitivity and ultra-low limits of detection. In this paper, several image analysis methods are evaluated for their ability to accurately enumerate single molecules bound to a microarray spot. Crucially, protein abundance in single cells can vary significantly and may span several orders of magnitude. This poses a challenge to single molecule image analysis. In order to quantitatively assess the performance of each method, synthetic image datasets are generated with known ground truth whereby the number of single molecules varies over 5 orders of magnitude with a range of signal to noise ratios. Experiments were performed on synthetic datasets whereby the number of single molecules per spot corresponds to realistic single cell distributions whose ground truth summary statistics are known. The methods of image analysis are assessed in their ability to accurately estimate the distribution parameters. It is shown that super-resolution image analysis methods can significantly improve counting accuracy and better cope with single molecule congestion. The results highlight the challenge posed by quantitative single cell analysis and the implications to performing such analyses using microarray based approaches are discussed.

## Introduction

The need for quantitative information in systems biology has been the driving force of a number of analytical techniques over the last decade or so, seeking to provide a fully resolved description of the underlying mechanisms at play. To quantify absolute numbers of biomolecules present in cells presents an enormous challenge. Microarray technology has become a popular tool for highly parallel analyses of biological markers; arrays of DNA, RNA, proteins, peptides, carbohydrates and other biomolecules are powerful research tools and play a critical role in biochemistry and molecular biology research. Microarrays are typically formed upon planar substrates, such as a surface modified glass slide, comprising thousands of small spots, typically on the order of 100 µm in diameter, where they react with specific analytes in a complex solution to perform a miniaturised bio-molecular assay.

Fodor *et al*. lay the foundation for microarrays in 1991 by demonstrating a process to build an array of synthesised peptides by sequentially depositing amino acid groups on a glass surface^1^. The substrate was functionalised using a combination of photolabile chemical building blocks and photomasks to synthesise an array of 1024 peptides. The technology was subsequently used to construct oligonucleotide microarrays because of interest in genome sequencing; nucleic acid synthesis was well known and the high throughput offered by microarrays was appealing. Schena *et al*. used a robot to mechanically print spots of complementary DNA onto glass using a quill-type metal pin^2^. Indeed, Schena’s technique has become the most widespread technique today in the preparation of protein microarrays.

Once sequencing the human genome was complete, attention focussed on the proteome and methods to study it. The success of the microarray to genomic studies was not immediately replicated in protein microarrays owing to the chemical complexity and diversity of the proteins themselves – proteins have a broad diversity of solubility, tertiary structures and amino acid content. All aspects of protein microarrays, such as printing, surface chemistry, geometry and capture probes, have undergone significant development, and continue to do so.

It would take over a decade for single cell analysis by microarray to be reported. Today, several approaches to single cell protein analysis have been reported with the capability to sense and quantify protein species in a highly complex or congested sample requiring no pre-separation stage^3–6^. Microarrays are compatible with the microfluidic format and, indeed, benefits from the reduced volume environment in respect of the proportion of the capture analyte that is bound^7,8^.

A number of optical techniques have been used to detect surface bound targets and conventional methods predominantly employ fluorescence slide scanners^9^. Image processing of such microarray scan data requires an identification step to dissect an image into single spot sub-images, a segmentation step to identify spot shape and classify pixels as either foreground, belonging to the spot, or background, and a step to extract signal intensity, that may involve additional measures to limit non-biological variation of the data^10^. The estimation of true signal is difficult for low-intensity spots and the desire to extend dynamic range to analyse targets at or near the limits of detection motivated, in part, the development of single molecule detection methods; touted as the ultimate limit in microarray performance sensitivity.

High resolution single molecule approaches to analysing microarrays have been greatly aided by the adoption of evanescent wave-based techniques, such as total internal reflection (TIRF) microscopy^11–13^. Hirschfield was the first to exploit evanescent wave excitation to optically detect single molecules^14^. More thorough surveys of the field and how it developed may be found elsewhere^15–18^. A crucial milestone in the development of single molecule microarrays was achieved when Hesse *et al*. reported high resolution scanning over large scales at high speed^19^. They notably demonstrated an extended dynamic range using their methods, which significantly exceeded conventional microarray analysis.

Owing to their sensitivity and low limits of detection, single molecule microarray methods are well suited to quantifying cellular constituents free of the cell and the ability to detect single biomarkers in liquid samples, particularly blood, can accelerate the discovery and use of more sensitive diagnostics. The enumeration of oncogenic proteins expressed within cancer cells has been demonstrated using antibody-based capture spots and is capable of detecting a little as 21 proteins from single cells^4,20^. Shirasaki *et al*. employed single molecule detection to monitor in real-time the secretion of the cytokine interleukin 1β from a single cell^21^. Conventionally, such assays require washing steps to remove unbound material prior to imaging. Single molecule microarrays imaged by TIRF are able to simplify assays by avoiding such steps since only bound molecules are detected. Jain *et al*. showed that employing single molecule methods enables the study of individual protein complexes and can help provide information on complex stoichiometry^22^.

The identification of single molecules and their accurate enumeration is key in quantitative microarray analysis. Methods of spot detection in fluorescence microscopy have been well reported; Izeddin *et al*. presented a wavelet based algorithm that focussed on single molecule localisation^23^. Smal *et al*. performed a thorough survey of techniques and quantitatively evaluated single molecule image analysis methods for peak detection^24^. However, there is a paucity of reports that focus on the methods of image analysis for high-resolution single molecule microarray data. The work of Mureşan *et al*. is an exception to this, wherein they report the detection of single molecules based on undecimated wavelet transforms^25^. Using the detection results, an estimation of the number of single molecules per spot could be made and could further discriminate between specific and non-specific signal.

The determination of what constitutes a specific, or true, signal is not a simple task in single molecule imaging; issues such as low signal to noise ratio (SNR), background estimation, impurities and variations in fluorophore labelling all contribute to errors. Methods of image analysis to detect single molecules are continually being developed but are predominantly designed to analyse super-resolution cell microscopy data, whereby the focus is generally toward single molecule localisation. Isolated fluorophores may be localised to a precision well below that of the diffraction limit^26^; however, imaging single molecules in cells inevitably leads to the problem of congestion where the target density in some structures is such that single molecules are no longer individually resolvable. Some super resolution microscopy techniques exploit the stochastic nature of photo-activatable fluorophore switching to image a subset of fluorophores in an otherwise congested region; a complete molecular image is then built up over time. Single molecule localisation microscopy (SMLM) is a proven bioanalytical tool and relies just as heavily on switchable fluorophores as it does on powerful algorithms to identify and estimate the positions of single molecules. Many algorithms currently exist along with standardised data to benchmark implementations that can provide guidance on which is most suitable for a particular application^27,28^.

In this work, methods of image analysis are assessed in their ability to accurately determine the number of single molecules in high resolution images of microarray spots. Images were processed with wavelet based transforms to enhance single molecule contrast or to denoise images. A concern when performing SMLM is the efficient detection of a statistically sufficient number of molecules for analysis. This needn’t be 100% of all target molecules for SMLM whereas it certainly is the case when quantitatively enumerating targets bound to a microarray spot. With this in mind, a super-resolution peak fitting algorithm is also tested for its performance in analysing microarray images. To evaluate methods, synthetic datasets were generated with known ground truth, ranging from 100 to 1 × 10^7^ molecules bound to a spot, equivalent to a density of molecules of 1.27 × 10^−2^ μm^−2^ to 1.27 × 10^3^ μm^−2^. The density of single molecules per spot varied such that images are considered either to be i) non-congested, where single molecules are sparse; ii) semi-congested, where the degree of overlap is significant; or iii) congested, where the density of single molecules is high and are no longer individually distinguishable.

It has been recently shown that the underlying mechanisms of gene expression may be uncovered from the summary statistics of single cell distributions of protein abundance^29^. It is therefore important to measure these distributions with sufficient accuracy in order to capture subtle variations in behaviour as a cell or pathway is stimulated or dysregulated. Synthetic datasets were generated whereby the number of single molecules per spot corresponds to single cell distributions of protein abundance whose summary statistics are known. The distribution parameters of which served as the ground truth to which the estimated distributions derived from single molecule counts were compared. Finally, the image analysis methods are tested with experimental single molecule microarray data generated from a microfluidic platform for the quantification of proteins with single cell resolution.

## Results

### Single molecule image analysis

**Figure 1:**
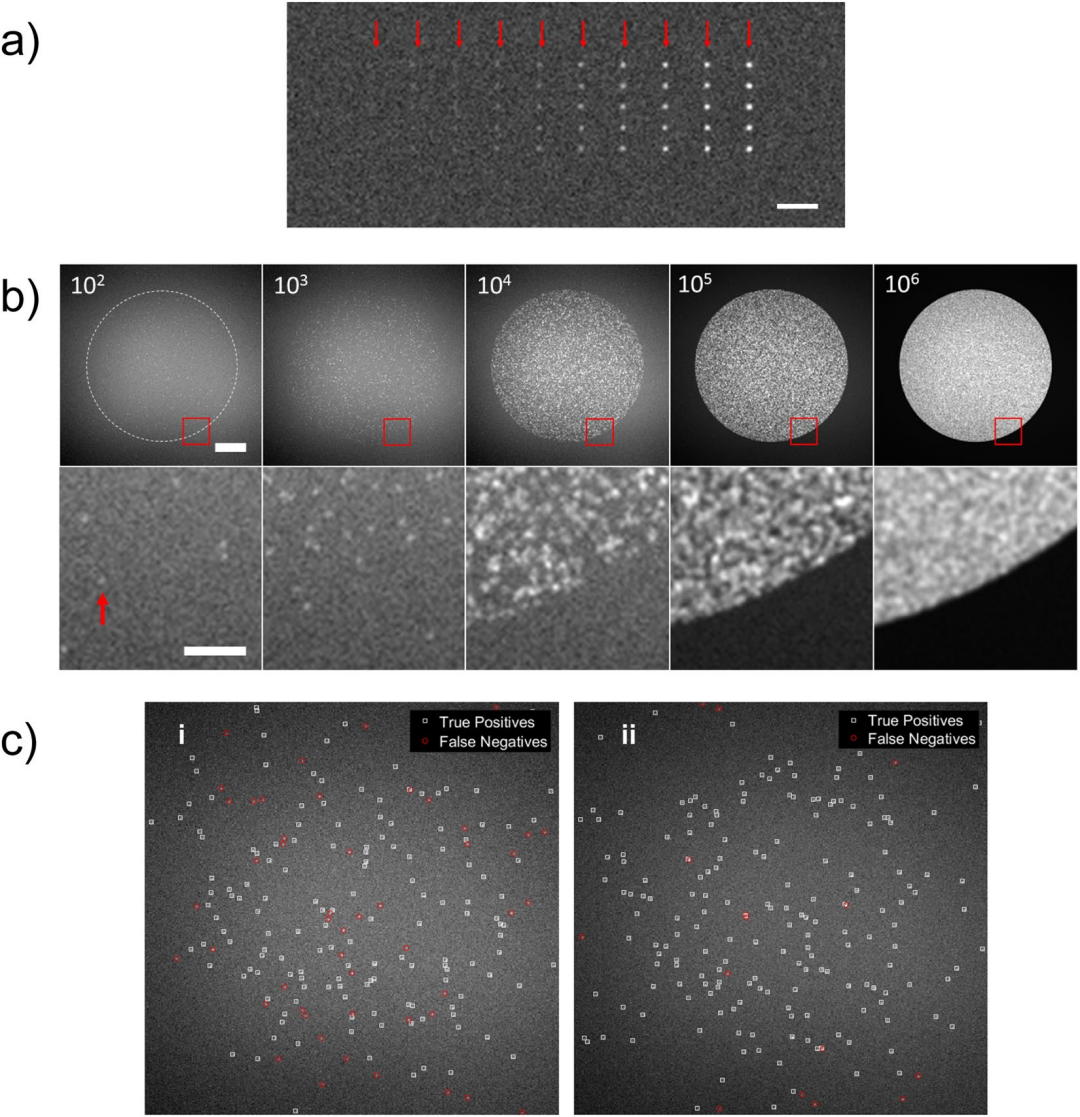
Synthetic single molecule image data. (**a**) The synthetic image shows a 10 × 5 test grid of single molecules. From left to right, the signal to noise ratio of the single molecules in each column is 1, 2.5, 3, 4, 5, 6, 7.5, 10, 12.5, 15 and 20. The red arrows indicate each column and helps to identify low signal to noise ratio single molecules. Scale bar is 5 μm. (**b**) Shown are exemplar images of simulated spots (SNR = 7.5). Each synthetic image is generated with a pre-defined number of single molecules randomly located inside a circular area which is intended to mimic a microarray spot. The labels indicate the number of single molecules on spot (10^2^ molecules per spot is equivalent to a molecule density of 1.27 × 10^−2^ μm^−2^) and the dashed circle in the leftmost image indicates the extent of the microarray spot area. The bottom row of images are zoomed in areas indicated by the red square in the top row. The scale bar for the top row of images is 20 µm, and 5 µm for the bottom row. (**c**) Images are processed using the image analysis methods under assessment to detect and enumerate the single molecules in each image; this is pre-defined and so the number of single molecules ‘counted’ by each method can be compared to the ground-truth to determine the accuracy as a function of analysis method, number of single molecules per spot and signal to noise ratio of the molecules. (i) and (ii) show exemplar results whereby the counting accuracy is <70% and >90%, respectively. The resolution of all images is 0.26 μm per pixel.

**Figure 2:**
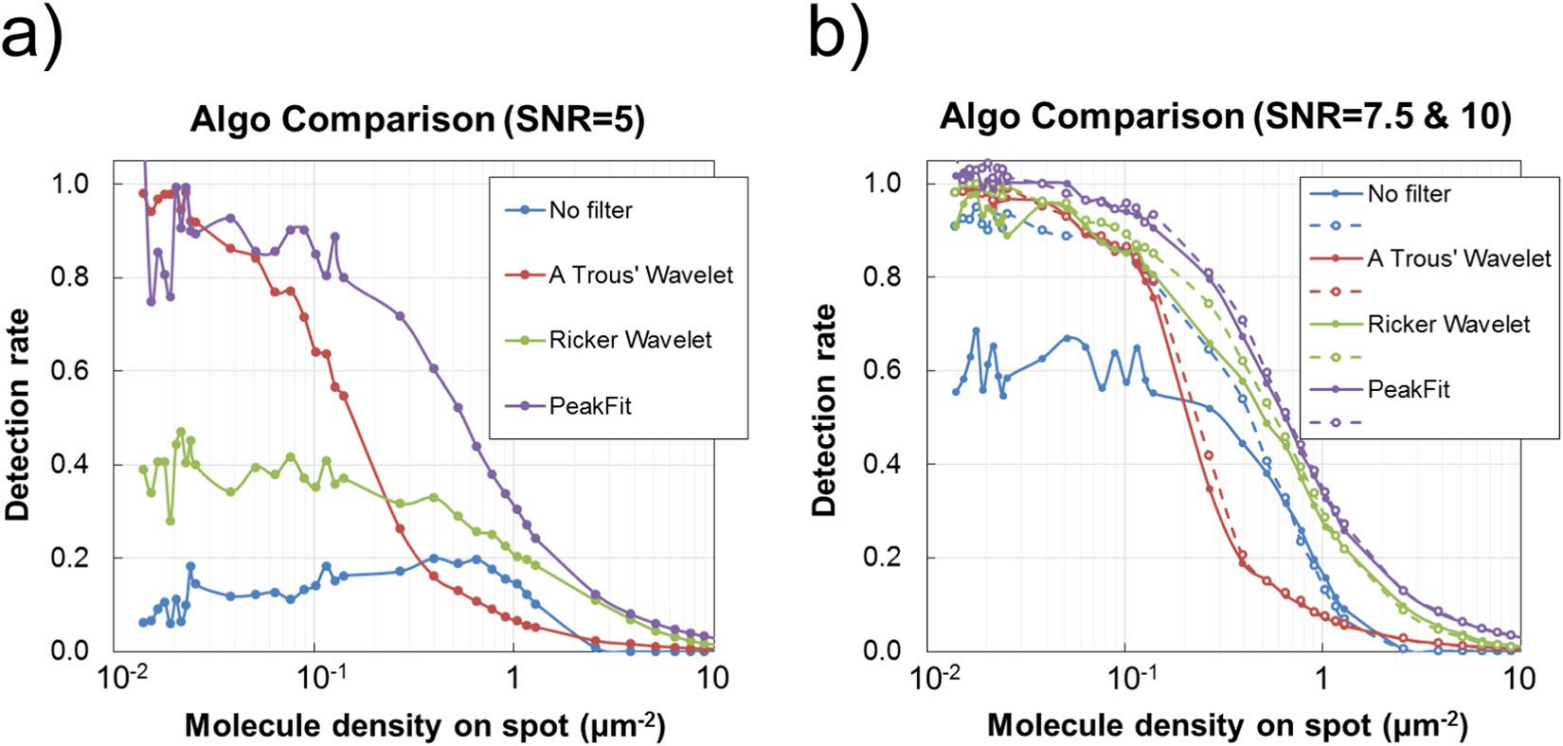
Non-congested arrays. The synthetic single molecule datasets are processed and the detection rate of each single molecule image analysis method, or algorithm for short, is assessed as the pre-defined number of single molecules in each image increases. Results show the performance of the algorithms to data with a signal molecule signal to noise ratio of (**a**) 5, (**b**) 7.5 (solid lines) and 10 (dashed lines). When the number of single molecules is low (<10^3^ per image), the density of the single molecules in the microarray spot is relatively low (<0.127 single molecules μm^−2^) and they can individually be distinguished. As the number of single molecules per images increases, so does their density on spot, such that the degree of overlap becomes significant and accuracy drops as a result. The figure labels ‘No Filter’, ‘A Trous Wavelet’ and ‘Ricker Wavelet’ represent the results of the intensity thresholding algorithms with either no filters or pre-processing, or pre-processing with the ‘à trous’ or Ricker wavelet transforms, respectively. Similarly, the ‘PeakFit’ figure label represents the results of the peak fitting algorithm.

The performance of the detection methods was quantitatively evaluated using synthetic image datasets (Fig. 1). Single molecule detection rate, a measure of accuracy, was determined as parameter settings were varied for synthetic images of varying image quality and number of single molecules. Additionally, methods were compared to intensity thresholding without any pre-processing to reduce noise or enhance signal. The synthetic image datasets were each characterised by a SNR of the single molecules; SNR values varied between 1 and 20. The detection rate is determined by comparing the ground truth to the count number produced using each image analysis method. How the detection rate varies for each algorithm to an increasing number of single molecules as a function of SNR is shown in Fig. 2, for SNRs of 5, 7.5 and 10. (The response to all SNRs is shown in Figure S2). For spots with a number of single molecules (N_SM_) that is low, their density is such that they are spatially well separated and individually distinguishable. Due to the limited resolution of the microscope, as the N_SM_ per spot increases, the likelihood of single molecules touching or overlapping also increases such that they are identified as connected components or single structures; thereby underestimating the counted N_SM_.

Intensity thresholding alone (No Filter) fails for SNR ≤ 5 and accuracy only exceeds 80% for SNR ≥ 10 for molecule density <0.127 (I_SM_ < 1000 molecules per spot) i.e. non-congested spots. Processing with either the Ricker (RW) or ‘a trous’ wavelet (ATW) significantly enhances the accuracy in single molecule identification for non-congested spots with low SNR and peak fitting results in the best overall performance; however, there are exceptions to this when comparing peak fitting to processing with an ATW for SNR ≤ 4 (Figure S2).

For SNR ≥ 10, all methods performed to a satisfactory degree for molecule density <0.127 (N_SM_ < 1000) where accuracy exceeded 82.1%. The accuracy profile of each method for SNR ≥ 10 as molecule density increases is typically sigmoidal; characterised by a fairly flat response up to 0.1 μm^−2^ (N_SM_ ~ 1000) beyond which accuracy reduces sharply such that it drops below 30% for molecule density> 1 μm^−2^ (N_SM_ > 10^4^), regardless of image analysis method. The point at which the accuracy of an algorithm begins to sharply reduce represents the degree to which it can identify overlapping single molecules as spots go from being non-congested to being semi-congested then congested. It is not surprising that the non-peak fitting methods perform similarly with regard to the point at which accuracy falls as spots become semi-congested.

### Analysing congested arrays

Protein abundance in single cells can span several orders of magnitude, and due to cellular heterogeneity this can arise even for cells in a genetically identical population. The complication to single molecule approaches when performing single cell analyses is that a sufficiently high cellular concentration of a protein in a subset of cells, for example, would result in some microarray spots becoming congested due to the amount of analyte captured. Modifying the density of surface sites in a microarray to maintain a regime whereby single molecules can be resolved has been previously suggested^30^. Therefore, unless the distribution of expected signal is sufficiently narrow, spots of varying density would be required on the same microarray and is not necessarily the most straightforward approach. Furthermore, a reduction in the density of surface sites will likely increase the limit of detection of a microarray assay, which is undesirable for single cell protein assays^7^. Single molecules could by selectively identified in congested microarray data by photo-activated localisation microscopy^31^ or by the detect and bleach strategy^20^. In the absence of more elaborate methods, an estimate of N_SM_ on congested microarray spots can be achieved by dividing the total integrated intensity by the average single molecule intensity. This strategy relies on the following experimental conditions: 1) the variation in the number of fluorescent labels per detection analyte is low; and 2) a low single molecule density, or non-congested, image may be acquired to accurately estimate single molecule intensity (I_SM_). Single molecules will accumulate on capture spots over time at a rate defined by the kinetics of binding. The second condition may be experimentally achieved by imaging spots as a function of time or, in order to minimise photobleaching, once at a sufficiently early time point and once when equilibrium has been achieved.

**Figure 3:**
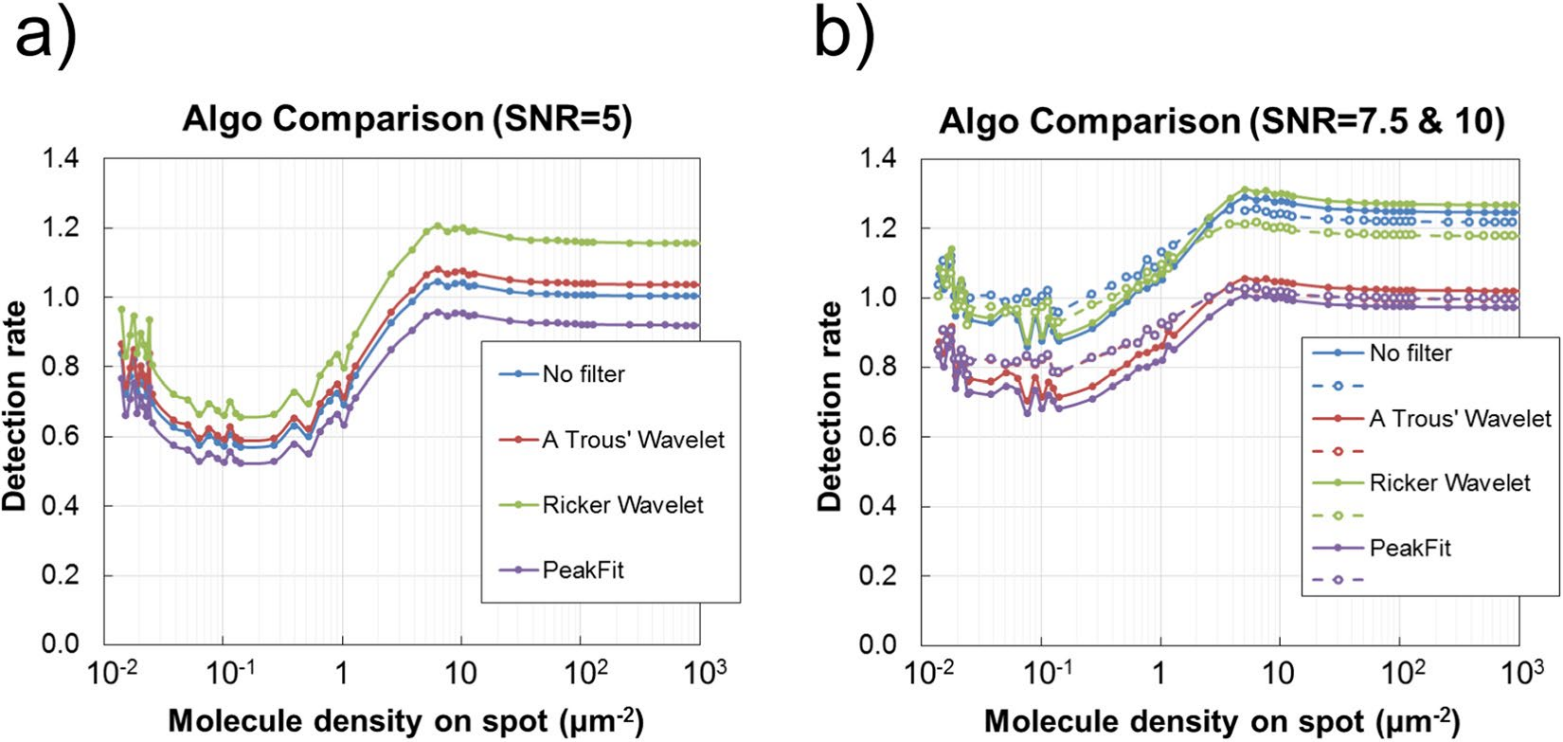
Congested arrays. In congested images, the number of single molecules is estimated by dividing the total array spot intensity by the average single molecule intensity, which is estimated using the single molecule algorithms described above on non-congested images. Results show the performance of the algorithms to data with a signal molecule signal to noise ratio of (**a**) 5, (**b**) 7.5 (solid lines) and 10 (dashed lines). The figure labels ‘No Filter’, ‘A Trous Wavelet’ and ‘Ricker Wavelet’ represent the results when using estimates of average single molecule intensity determined using the intensity thresholding algorithms with either no filters or pre-processing, or pre-processing with the ‘à trous’ or Ricker wavelet transforms, respectively. Similarly, the ‘PeakFit’ figure label represents the results when using an estimate of average single molecule intensity determined using the peak fitting algorithm.

Single molecule intensity is first estimated using the methods described above. For non-peak fitting algorithms, average single molecule intensity is estimated by summing thresholded pixel values and dividing by the number of identified peaks. For the peak fitting algorithm, each single molecule is fit to an isotropic 2D Gaussian profile which allows the full distribution of I_SM_ to be determined per image; depending on which is more appropriate, an average or median I_SM_ may be calculated. Images from the same datasets as above are intensity thresholded based on the variation of the background pixel intensity (see Methods); to estimate N_SM_, the sum of the surviving pixel values is divided by the average I_SM_. Figure 3 shows the detection rate of the ‘congested’ image analysis method when using such estimates of I_SM_ for SNR of 5, 7.5 and 10 (see Figure S3 for responses to all SNR).

The variation in detection rate relates to how the accuracy to which I_SM_ is estimated and the relative contributions of the background and single molecules to pixel intensity when summed across the image. For low SNR, intensity thresholding tends to identify more intense single molecules, systematically discriminating against low intensity molecules thus positively skewing the distribution. In absolute terms, the total single molecule intensity of these identified molecules is itself underestimated since there will be a proportion of the signal below threshold that is lost. Of course, the process of thresholding is a balance between the rejection of background and preservation of signal intensity. As confidence in background rejection increases (related to *n*) so does the amount of true signal which is undesirably rejected. This poses less of a problem for digital counting but becomes an issue for congested arrays as we must rely on an accurate estimate of single molecule intensity.

It is evident from Fig. 3 that methods of analysis for congested spots were also applied to non-congested spots (molecule density <1 μm^−2^, N_SM_ <10^3^). While in practice this would not be sensible, it does help us to understand how inaccuracy arises in semi-congested and congested spots. For non-congested spots, the true count is predominantly underestimated. In these spots, the density of single molecules is low and the relative contribution of the background is high. Portions of each single molecule that fall below the threshold are discarded leading to an underestimation of the total signal intensity on spot (I_spot_). This, in addition to the overestimation of the average I_SM_, leads to an overall underestimation of N_SM_ per image, which is consistent for all algorithms analysing non-congested spots. As single molecules accumulate, they will begin to raise the average intensity across the spot. At some point the average intensity will exceed the threshold intensity. As single molecules are further ‘added’ to the spot, no proportion of a single molecule’s total intensity is any longer discarded by thresholding and the increase in total spot intensity is equal to the total intensity of the additional single molecule. As N_SM_ increases in this regime, the detection rate will increase and accuracy improves. For very high N_SM_, the proportion of the thresholded intensity to the total spot intensity decreases and the detection rate will plateaux. The detection rate will tend toward a value which is the accuracy to which I_SM_ is estimated.

In the cases of the intensity thresholding alone (No Filter) and the RW non-fitting algorithms, the number of molecules may be overestimated in some cases. This would arise due to the relative degrees by which I_spot_ and I_SM_ are under- and overestimated, respectively. This is suppressed when using the ATW as the process denoises images and the effects due to the relative contribution of background intensity are suppressed. Peak fitting allows the 2D Gaussian profile of the single molecule to be known and therefore more reliably estimate the I_SM_. For SNR ≥ 10 the accuracy ‘profile’ flattens due to the relative contribution of the noise and any errors arising from thresholding becomes relatively small.

### Accurately measuring single cell distributions

**Figure 4:**
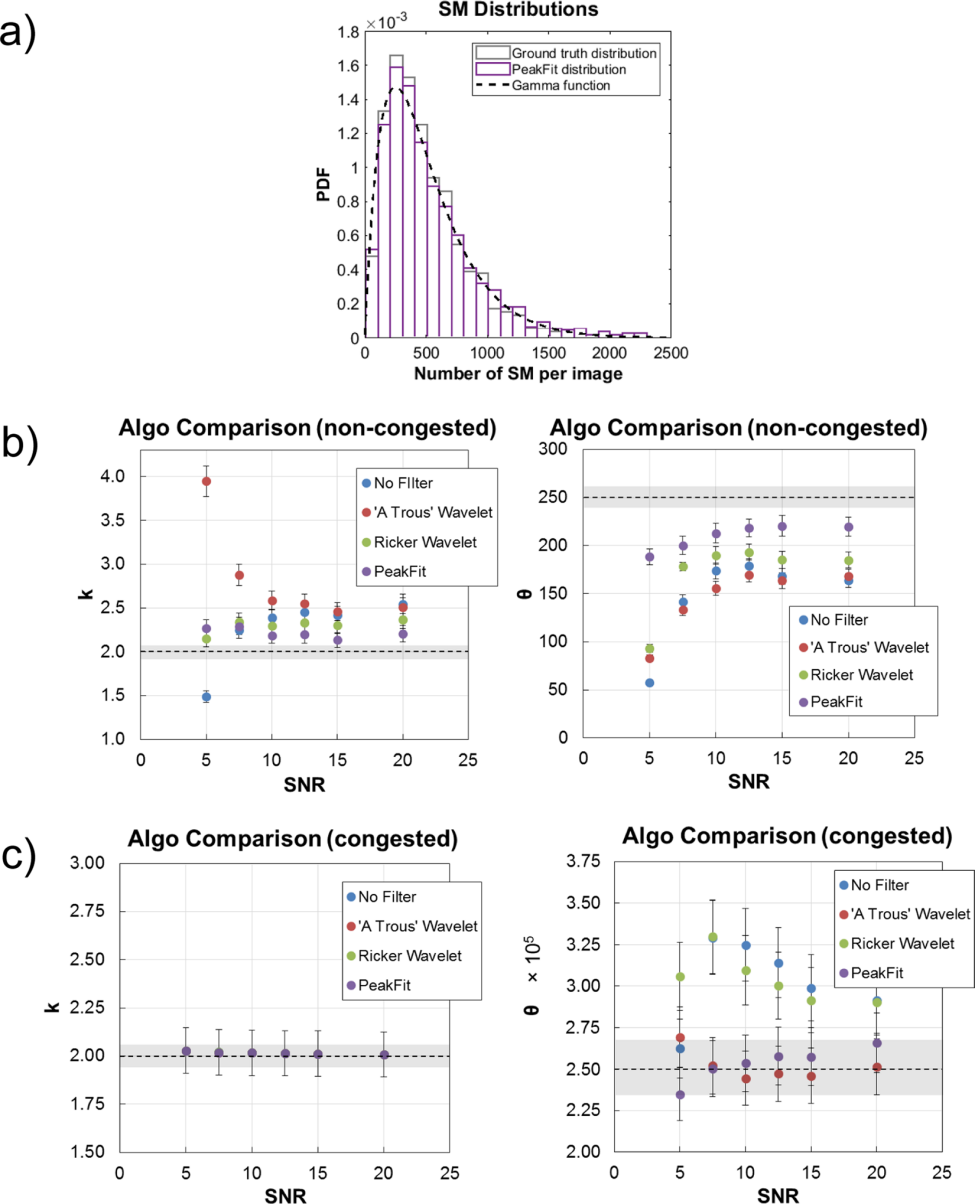
Synthetic data to model single cell distributions. Synthetic image datasets are generated whereby the number of single molecules in the microarray spot follows an asymmetric gamma distribution. (**a**) Shown are the probability density function of histograms comparing the ground truth distribution (grey edges) of pre-defined single molecule number to the single molecule counts estimated using the peak fitting algorithm (purple edges). Overlaid is the continuous gamma function (dashed black line; k = 2.0, θ = 250) from which the ground truth distribution and image datasets are generated. The parameters tested here are such that the gamma distribution is peaked in the (**b**) non-congested (k = 2.0, θ = 250) or (**c**) congested (k = 2.0, θ = 2.5 × 10^5^) regimes. Images were processed to estimate the number of single molecules, the distribution of which was fitted to estimate the parameters. The horizontal dashed black line indicates the ground truth parameter value. The error bars for each data point indicate the standard error of fitting. The grey rectangular region vertically centred on the dashed line indicates the standard error of the fit to the ground truth distribution values.

**Figure 5:**
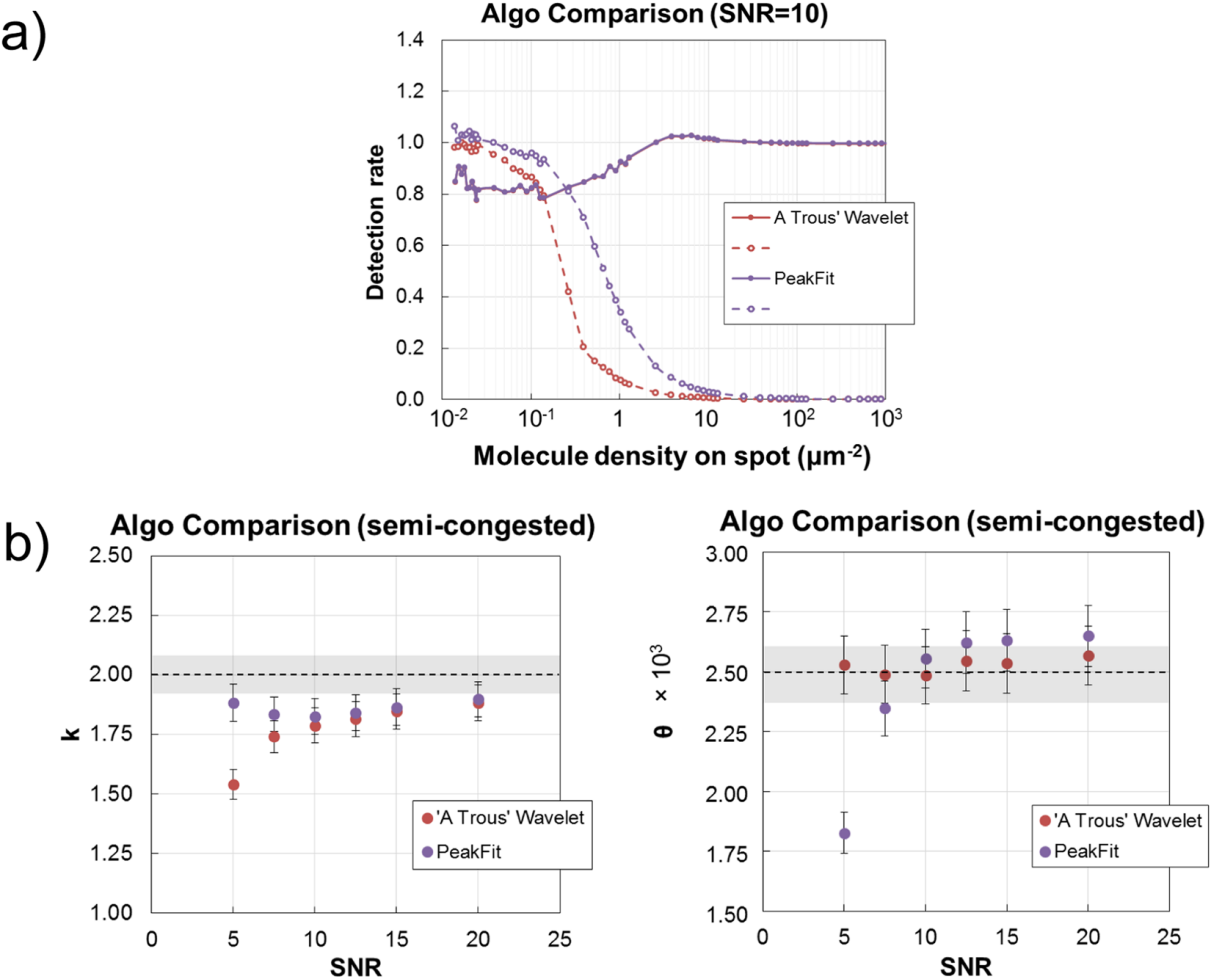
Synthetic data to model single cell distributions. Synthetic datasets are generated whereby the number of single molecules in the microarray spot follows a gamma distribution with parameters such that it is peaked in the semi-congested (k = 2.0, θ = 2.5 × 10^3^) regime. (**a**) Image analysis methods best suited to either non-congested or congested data were combined to maximise accuracy in this regime. (**b**) Results show the accuracy in estimating distribution parameters when using the ‘à trous’ wavelet transform and peak fitting image analysis algorithms. The horizontal dashed black line indicates the ground truth parameter value. The error bars for each fit indicate the standard error of fitting. The grey rectangular region vertically centred on the dashed line indicates the standard error of the fit to the ground truth distribution values.

Single molecule microarrays are well suited to quantitative single cell analysis. Single cell protein abundance in a population of cells is well known to vary and datasets will reflect this information as a variation in the number of single molecules per microarray spot. Such data is important for models of gene expression^29,32^ and therefore an accurate measure of this variation is obviously desirable. With this goal in mind, synthetic datasets were generated whereby the number of single molecules per spot within a dataset followed a gamma distribution with known shape and scale parameters. The gamma distribution model is well known to faithfully describe cell to cell variation in protein expression^33,34^. The distributions were formed by the same shape parameter (k = 2.0) and their scale parameter varied such that the distributions were peaked in the non-congested (θ = 250 SMs, 3.18 × 10^−2^ μm^−2^), semi-congested (θ = 2.5 × 10^3^ SMs, 0.318 μm^−2^) and congested (θ = 2.5 × 10^5^ SMs, 31.8 μm^−2^) regimes. For each of the 3 distributions, datasets were generated with different SNR, which varied from 5 to 20 (Figs 4 and 5).

As a control to test the competence of the fitting procedure, the ground truth distribution of known N_SM_ is fitted alongside the counted N_SM_ (Fig. 4a). The shape and scale parameters were estimated by fitting for all distribution datasets. For the non-congested datasets, it was possible that the counted distribution qualitatively resembled the ground truth distribution. However, the fitting parameters of these distributions would numerically differ significantly from the input ground truth parameters. In general, parameter estimates become more accurate as SNR increases; however, for non-congested data there is a tendency to overestimate or underestimate the shape and scale parameters, respectively (Fig. 4b).

Here it is worth noting the relative performance of each algorithm in estimating N_SM_ (Figs 2 and 3) and in producing data to estimate the parameters of a distribution (Fig. 4b and c). When estimating the summary parameters of a gamma distribution, accuracy in the shape parameter may in some cases be prioritised over accuracy in the scale parameter. Errors in scale will arise predominantly to the counting accuracy deviating from 100%, whereas errors in shape will arise predominantly to non-linear ‘response’ in detection rate as evident in Figure S2 for low SNR. This is exemplified by the shape parameter being best estimated when the distribution is peaked in the congested regime (Fig. 4c); an inspection of the detection rate of the analysis of congested arrays (Fig. 3) shows a ‘flat’ response over the range of values associated with the distribution (k = 2.0, θ = 2.5 × 10^5^). Similarly, an inspection of the detection rate of the single molecule analysis methods of non-congested arrays does show a ‘flat’ response, albeit a shallow negative gradient (Fig. 2c). These features are able to explain the apparent bias observed when fitting non-congested data (Fig. 4b). It has been shown that in the case of single cell protein analysis, microarrays may be calibrated using standard curves established using known concentrations of recombinant proteins. Such calibration curves would factor out any errors in scale but would not be capable of correcting errors in shape^20^. This exemplifies the necessity for a high degree of accuracy over a wide dynamic range.

Analysing synthetic data distributed in both the non-congested and congested regimes is straightforward whereas semi-congested data is more challenging as it is the region in which neither image analysis approach is best suited. This is exemplified in Figure S4, where the analysis of such data fails to faithfully reproduce the ground truth distribution. There is a propensity for the intensity thresholding alone (No Filter) and the RW non-fitting algorithms to perform poorly in non-congested images at low SNR and to generally overestimate N_SM_ on congested spots at SNR > 5. Despite this, their estimation of spot and average single molecule intensity results, serendipitously, when calculating N_SM_ from total I_SM_, in more accurate estimations in non- and semi-congested images. An argument based on the overall performance of both these approaches allows us to proceed in the enumeration of semi-congested data with the ATW non-fitting and peak fitting image analysis methods, only. The performance of the single molecule and congested image analysis methods were compared as the number of single molecules per spot was increased through each of the 3 regimes, noting the point at which the performance of the latter would exceed the former (Fig. 5a). This crossing point would help decide what image data is analysed with which method and is dictated by the density of single molecules on spot and the degree to which they are no longer individually distinguishable. In practise this may be difficult to determine without some measure of the proximity of single molecules to one another. Here, with the data presented it is justifiable to simply determine the numerically higher count as the more precise (Fig. 5a) and represents a, somewhat, best case scenario when applying these methods to real data. The semi-congested results in Fig. 5b show that the combined approach enables an estimation of distribution parameters with ~90% accuracy for shape (ATW SNR ≥ 5) and scale (PeakFit SNR ≥ 5).

The effect of varying the scale parameter and determining the response to other distribution types was not tested.

**Figure 6:**
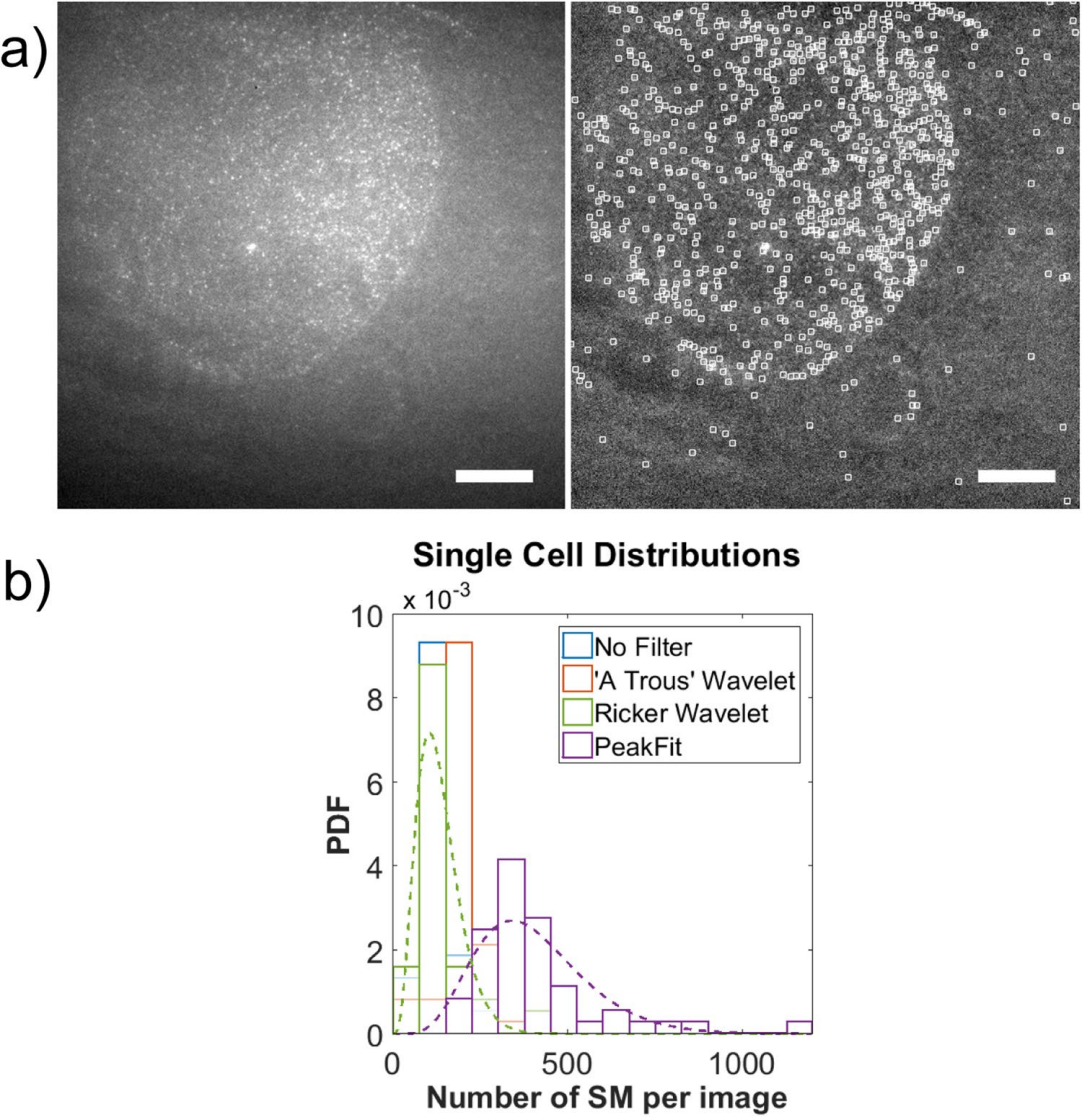
Testing experimental single cell data. (**a**) (i) Exemplar high-resolution image (0.26 μm per pixel) centred on a single microarray spot. Fluorescent single molecules represent the capture antibody–protein–detection antibody complex of a sandwich assay. The protein is p53 captured from MCF7 cells using a microfluidic based method of single cell analysis using single molecule microarrays. (ii) Shown is the image in (i) processed with an overlay of detected single molecules. Scale bars are 20 μm. (**b**) The results of single cell pulldowns (n = 50) are analysed. Shown are the probability density function of histograms of the single molecule counts estimated using the image analysis methods under test. The dashed green and purple lines are fits of a gamma function to the histograms produced using the intensity thresholding algorithm (pre-processing with the Ricker wavelet transform) and the peak fitting algorithm, respectively.

Single cell data was acquired using a microfluidic based single molecule microarray method^4^. MCF7 cells are removed from culture and a single cell suspension is created. Cells are flowed into a microfluidic device and isolated into individual analysis chambers; the chip used here comprised of 50 analysis chambers wherein a single antibody capture spot is located. The protein p53 was captured using a suitable capture antibody. Since p53 is unlabelled, a second detection antibody labelled with a fluorophore is present in the solution which binds to free p53 or the immobilised capture antibody-p53 complex at the surface. Spots are imaged by TIRF microscopy 90 min after cells are optically lysed to ensure binding equilibrium had been reached. The basal expression of p53 protein in MCF7 cells is such that captured protein on each spots typically results in a molecule density of <0.1 μm^−2^ and therefore suitable for the non-congested methods of analysis detailed above. Figure 6 shows the resultant distributions of counted single molecules from cells using each of the image analysis methods. The distribution of the counts using peak fitting algorithm is clearly different than those produced by the intensity thresholding based methods. For example, the counted distribution of single molecules as a result of the RW non-fitting (k = 4.80 ± 0.94, θ = 27.9 ± 5.7) and peak fitting algorithms (k = 6.51 ± 1.30, θ = 62.0 ± 12.8) are fit by a gamma distribution. Numerically, and visually by inspection of the fits in Fig. 6, these are clearly different counted single molecule distributions. The ground truth is unknown for the single cell single molecule image data and therefore an absolute measure of accuracy for each of the image analysis methods is not possible. The results from analysing the synthetic datasets would suggest that the peak fitting algorithm is likely to be the most accurate. By testing these methods of image analysis on experimental data with unknown ground truth the necessity is further highlighted for a high accuracy to be maintained over a range of molecule densities on spot.

## Discussion

The performance of a set of image analysis methods has been quantitatively assessed as applied to single molecule microarrays. Their performance is assessed using realistic synthetic data with known ground truth over a wide dynamic range and as SNR is varied; the absolute values of which are applicable to single cell protein analysis. This study has highlighted the importance of accurate single molecule enumeration when analysing data upon which quantitative models of systems biology increasingly relies. Synthetic datasets which serve to model the wide dynamic range of single cell protein expression and cellular heterogeneity were analysed. The image analysis method involving peak fitting performed with the highest accuracy, overall.

The results presented here show that peak fitting can maintain a detection rate in excess of 80% for molecule densities approaching 0.3 μm^−2^ and with the accurate estimate of single molecule intensity can effectively exceed 10^3^ μm^−2^ using the methods above. A number of approaches have been reported based on their ability to cope with high density data. Originally used in astronomy to analyse crowded stellar fields^35^, methods that fit populations of single molecules with multiple PSFs instead of just one can improve accuracy when detecting overlapping molecules. Holden *et al*. implemented this in DAOSTORM^36^. Zhu *et al*. reported a sparse recovery technique using compressed sensing and showed this could work with much higher molecule densities when compared to DAOSTORM^37^. In summary, single molecule fitting, DAOSTORM and compressed sensing were capable of maintaining a detection rate of 80% or higher up to densities of ~0.5 μm^−2^, ~2 μm^−2^ and ~10 μm^−2^, respectively, as estimated from published results using super resolution imaging systems^36,37^.

The density of single molecules is an important consideration to their reliable detection on microarray spots. While strategies to alleviate this, such as the reduction of binding site density, have been proposed they are not compatible with maintaining the requisite sensitivity necessary for measuring low abundance proteins in single cells^7^. Certainly, the digitisation of peak detection enables single molecule counting to be more robust to systematic variation. The accuracy of congested data is more prone to variations in systematic parameters such as laser intensity, emission collection efficiency, detection camera, amongst other factors. Of course, the accuracy of data in congested regions depends on the accurate measurement of single molecule intensity. The results quantitatively show how this affects the accurate estimate of the number of single molecules bound to a congested spot. It must be noted that in generating synthetic datasets, quenching was not taken into account and may be an issue for microarray spots with sufficiently high binding site density.

An effective alternative to reduce congestion, would be to adopt the use of photo-switchable fluorophores and super-resolution microscopy methods such as photo-activated localisation microscopy (PALM) or stochastic optical reconstruction microscopy (STORM). Although, if appropriately implemented, such methods are in principle capable of effectively reducing imaged molecule density, there are a number of non-trivial challenges which limit the accurate estimation of single molecule numbers. These include photobleaching, fluorophores which are never activated and distinguishing the observation of the same fluorophores in multiple consecutive or non-consecutive frames. The detect and bleach (DAB) method has also been previously described to be capable of regaining single molecule counting in densely occupied spots^20^. DAB works by differencing subsequent frames during continuous imaging of the spot and in the regime whereby molecular arrival or bleaching rate is low, single molecules which bind and others that are irreversibly bleached will be sparse and individually identifiable.

There are ongoing public challenges that exist to evaluate and benchmark new and existing techniques, with particular focus on localisation microscopy^28^. The methods of image analysis analysed here were assessed with quantitative single cell analysis in mind.

## Methods

### Single molecule detection

#### Data synthesis

Simulation images are generated with randomly located single molecules inside and outside a circular area which demarcates the microarray spot area. Each generated dataset contains 65 512 × 512 pixel images: of which 55 are 512 × 512 pixel images that contain 10–10^7^ randomly located on-spot single molecules in addition to 100 randomly distributed single molecules; a further 10 images contain 100 randomly distributed single molecules only and serve as background, or negative control, images. 11 datasets were generated with varying signal to noise ratios (1, 2.5, 3, 4, 5, 6, 7.5, 10, 12.5, 15 and 20). Each image within a dataset are independent and represent different spots in a microarray. Each dataset is characterised by a different SNR and is generated independently so the pre-defined number of single molecules in each image have different coordinates.

Synthetic image parameters were selected to correspond with the microscope system reported in reference^4^. To begin, the point spread function of the system was measured by imaging 100 nm fluorescent beads (Invitrogen Molecular Probes, FluoSpheres 540/560). Each simulated single molecule was generated using a 2D isotropic Gaussian function where the width corresponds with the measure point spread function (equivalent to 1.6 pixels in the 512 × 512 pixel image).

The resultant synthetic images are 512 × 512 pixel but to begin with a higher ‘super’ resolution 2560 × 2560 pixel image is generated. Uniformly distributed random numbers were used to generate single molecule coordinates in each microarray spot in an image. The pixel intensity of the super resolution image was then binned using a square 5 × 5 binning kernel to produce a 512 × 512 image. Larger binning kernels may be used to down sample images to lower resolutions to simulate other microarray imaging systems^25^. Our single molecule imaging system incorporates objective-type TIRF so noise such as that from immersion oil autofluorescence and scatter from the excitation laser are taken account of by adding a mean background intensity to each pixel. The excitation intensity profile of our TIRF microscope system was imaged and normalised using the peak intensity. The simulated spot images were then multiplied by the normalised laser profile image to better simulate real data. Poisson noise was then applied. The signal-to-noise ratio (SNR) of the single molecules was defined as *SNR* = (*S* − *B*)/*σ*, where *S* is the peak single molecule pixel intensity and *B* and *σ* are the average and standard deviation of background pixel intensity, respectively.

To better capture systematic variations in microarray performance, spot diameter and capture density may be varied to experimentally determined degrees. Here, to focus on errors arising from image analysis, the simulated data is generated with no intra- or inter-spot variation in microarray spot quality. Microarray spots of constant 100 μm diameter are modelled. The modelled microarray spot is centred in a 512 pixel × 512 pixel image, with pixel dimensions equivalent to a physical area of ~133 μm × 133 μm.

### Synthetic single cell datasets

The resulting distribution when measuring the protein abundance per single cell in a population of cells approximates the steady state protein abundance. To simulate single molecule microarray data from single cell experiments, images were synthesised as above whereby the number of single molecules on-spot were randomly generated from distributions with defined summary statistics. Datasets were generated whereby asymmetric gamma distributions with identical shape parameters (k = 2.0) are peaked in the non-congested (θ = 250 SMs), semi-congested (θ = 2.5 × 10^3^ SMs) and congested (θ = 2.5 × 10^5^ SMs) regimes and were used to assess the counting algorithms performance in faithfully measuring these distributions.

### Image Analysis

In summary, original images are field flattened, filtered then thresholded to produce images from which peak counting is performed. The methods employed here are computationally fast and datasets are typically processed within a few minutes.

Image filtering was performed using either the ‘à trous’ or Ricker wavelet transforms, which help to denoise images and enhance the signal. The ‘à trous’ wavelet-based filtering technique is a 2D isotropic undecimated wavelet transform^38^. The algorithm was implemented using the ‘ATrousJ_Filter’ plugin by Ihor Smal^24^ for ImageJ/Fiji^39^ using a B-spline scaling function of third order. Each image is decomposed into planes of wavelet coefficients (see Figure S1). The first plane contains high frequency components and the plane containing most of the noise. The second and third planes contains structures best associated with single molecules whereas higher order planes contain increasing lower spatial frequencies and are better associated with coarser image details.

The Ricker wavelet, also known as the Mexican hat wavelet owing to the shape of its 2D processing kernel, is the application of a Laplacian of Gaussian to a 2D image (see Figure S1). This type of edge detection kernel is an established method for object detection, such as single molecules and biological cells^40,41^. The algorithm was implemented using the ‘Mexican Hat Filter’ plugin by Dimiter Prodanov for ImageJ/Fiji.

Single molecules were either detected by intensity thresholding or by fitting peaks using a 2D Gaussian function. When intensity thresholding, pixel values were set to zero for pixels whose intensity value was less than *μ* + *nσ*, where *μ* and *σ* are the mean and standard deviation of background pixel intensity, respectively, and *n* is an integer which defines the probability that a pixel ‘belongs’ to a single molecule. Pixels that survive thresholding are then retained based on cluster size, 4–36 pixel^2^, and circularity, greater than 0.5. The number of single molecules is the number of remaining particles and the average single molecule intensity can be estimated.

To perform 2D Gaussian peak fitting, algorithms were implemented using the GDSC plugin by Alex Herbert^42^ for ImageJ/Fiji. A PSF estimator first determines by optimisation the average Gaussian parameters of the single molecules from intensity peaks in the dataset by least squares fitting. All peaks in each image are subsequently tested against this Gaussian profile and only counted as single molecules if they fall within a precision threshold for size and intensity. The total number of single molecules is simply the number of peaks in the image which converge to the 2D Gaussian profile.

For images where the density of single molecules results in a significant degree of overlap, single molecules become difficult to individually distinguish. The number of single molecules is then estimated from dividing the total intensity of a thresholded image by the average single molecule intensity.

### Single Cell Analysis

#### Cell culture

MCF7 (ATCC) cells were cultured using high glucose Dulbecco’s Modified Eagles Medium (DMEM; ThermoFisher Scientific, UK) supplemented with 10% (v/v) foetal bovine serum (FBS; ThermoFisher Scientific, UK) in polystyrene flasks in a 5% CO_2_ 37 °C cell incubator. Suspensions of single cells are prepared by detaching cells in culture using Accutase solution (Sigma, UK).

#### Single cell microarrays

A microfluidic based method was used to facilitate single cell protein analysis based on previously reported work^4^. The PDMS microfluidic chip is fabricated using well known methods of soft-lithography^43^. It is formed of a main channel through which single cell suspensions are flowed to which an array of analysis chambers (n = 50) are connected. Each analysis chamber has dimensions 300 μm × 300 μm × 35 μm resulting in an assay volume of 3.15 nL. The chip is sealed using a functionalised coverslip (Nexterion; Schott, Europe) upon which an anti-p53 antibody (Enzo Life Sciences, UK) microarray is printed using an OmniGrid Micro microarrayer (Digilab, UK). The microarray spotting solution contained the anti-p53 capture antibody mixed 1:1 with a print buffer formed of 3 × saline-sodium citrate buffer, 1.5 M betaine supplemented with 0.01% SDS; the final concentration of antibody in the spotting solution was 0.5 mg mL^−1^. The spots are printed at defined locations which allowed them to be aligned to the PDMS chip such that a single antibody spot is aligned to the centre of each analysis chamber. The chip is filled with a solution of 0.25 μg mL^−1^ fluorescent detection antibody (anti-p53 DO1 labelled with Alexa Fluor 488; Santa Cruz, USA) with 4% bovine serum albumin in phosphate buffered saline.

#### Platform and procedure

The platform has been described in detail elsewhere^4^. An inverted microscope (Nikon Ti-E; Nikon, Japan) forms the basis of the experimental platform. Single cells are individually isolated into analysis chambers using an optical trap (1070 nm YLM-5 Ytterbium fibre laser; IPG Photonics, UK). Once all analysis chambers are occupied, single cell lysis is achieved optically by launching a high energy laser pulse (6 ns pulse 1064 nm Surelite SLI-10 Nd:YAG; Continuum, USA) into the medium immediately surrounding the cell. The pulse produces an expanding cavitation bubble which shears the cell membrane allowing intracellular contents to be released. Antibody spots are imaged using objective-based total internal reflection fluorescence (TIRF) microscopy (488 nm Versalase; Laser 2000, UK) and an electron-multiplied CCD camera (IXON DU-897E; Andor Technologies, Ireland). The acquired images are analysed using the methods above.

## Electronic supplementary material

Supplementary Info
