# Supplementary material for: Evaluating single molecule detection methods for microarrays with high dynamic range for quantitative single cell analysis
Source: Sci Rep. 2017 Dec 20;7:17957. doi: 10.1038/s41598-017-18303-z (PMC5738400; doi:10.1038/s41598-017-18303-z)
Supplement: Supplementary file 1 — Supplementary Info [file 41598_2017_18303_MOESM1_ESM.doc]

**Evaluating single molecule detection methods for microarrays with high dynamic range for quantitative single cell analysis**

Ali Salehi-Reyhani

Dept. Chemistry, Institute of Chemical Biology, Imperial College London, London, SW7 2AZ

**Supplementary Information**

| 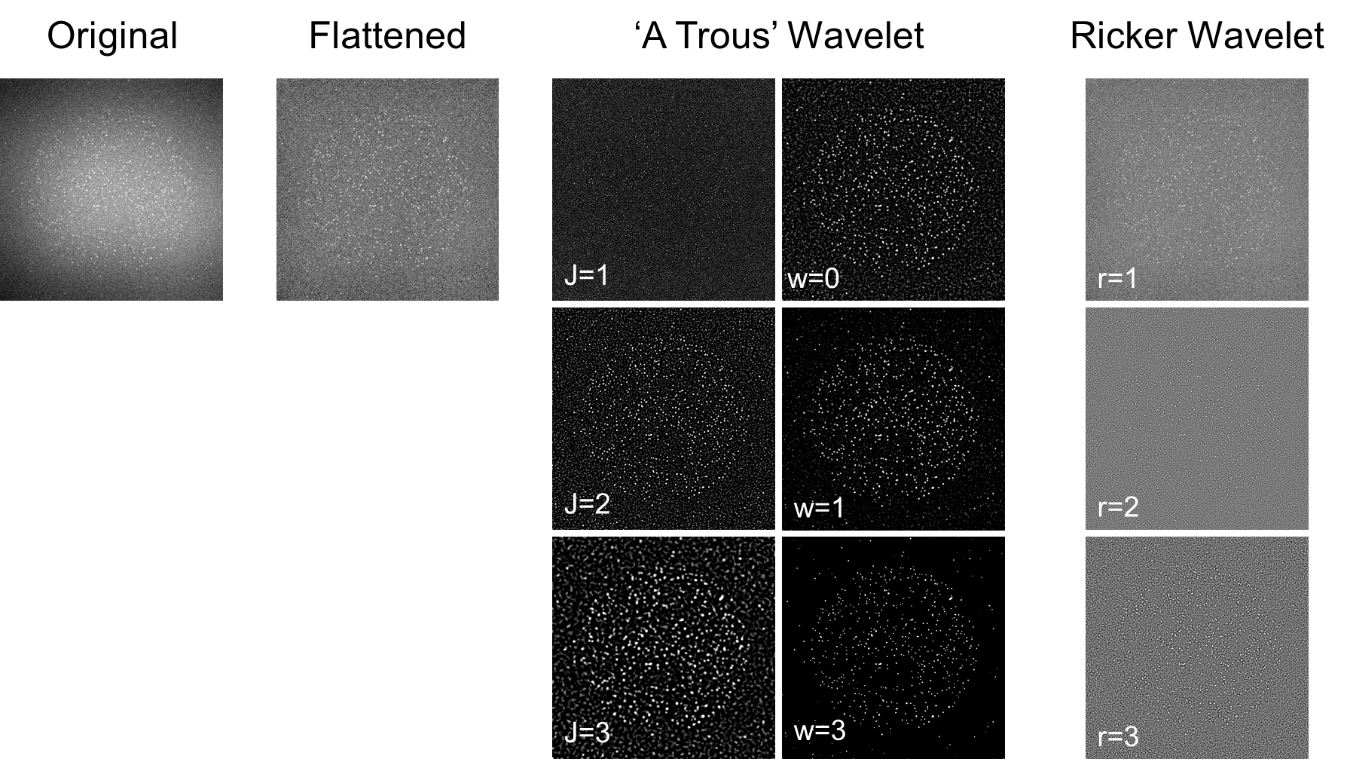 |
| --- |
| **Figure S1:** Synthetic images are processed using image analysis methods, which are assessed for their accuracy in enumerating single molecules within a microarray spot. The panel of images provides examples of the effect of flattening images and processing with the ‘à trous’ or Ricker wavelet transforms. Flattened - flattening images removes or suppresses the variation in image intensity as a result of the intensity profile of excitation light, typical of total internal reflection microscopy. ‘A Trous’ Wavelet - wavelet planes of scale *J* are shown for the flattened image. Low J value planes correspond to higher frequency components or spatial variations over smaller length scales and the plane containing the most of the image noise is *J*=1. Higher J value planes correspond to lower frequency components or spatial variations over longer length scales. The wavelet threshold (*w*) is set to discard wavelet coefficients of low amplitude associated with the noise. Ricker Wavelet – a ‘Mexican hat’ kernel of radius r is convolved with the image which performs smoothing and edge detection. A smaller radius better preserves single molecule edges but results in lower noise reduction; whereas, a larger radius is better at denoising but can smear single molecule edges and hinder their detection. |

| 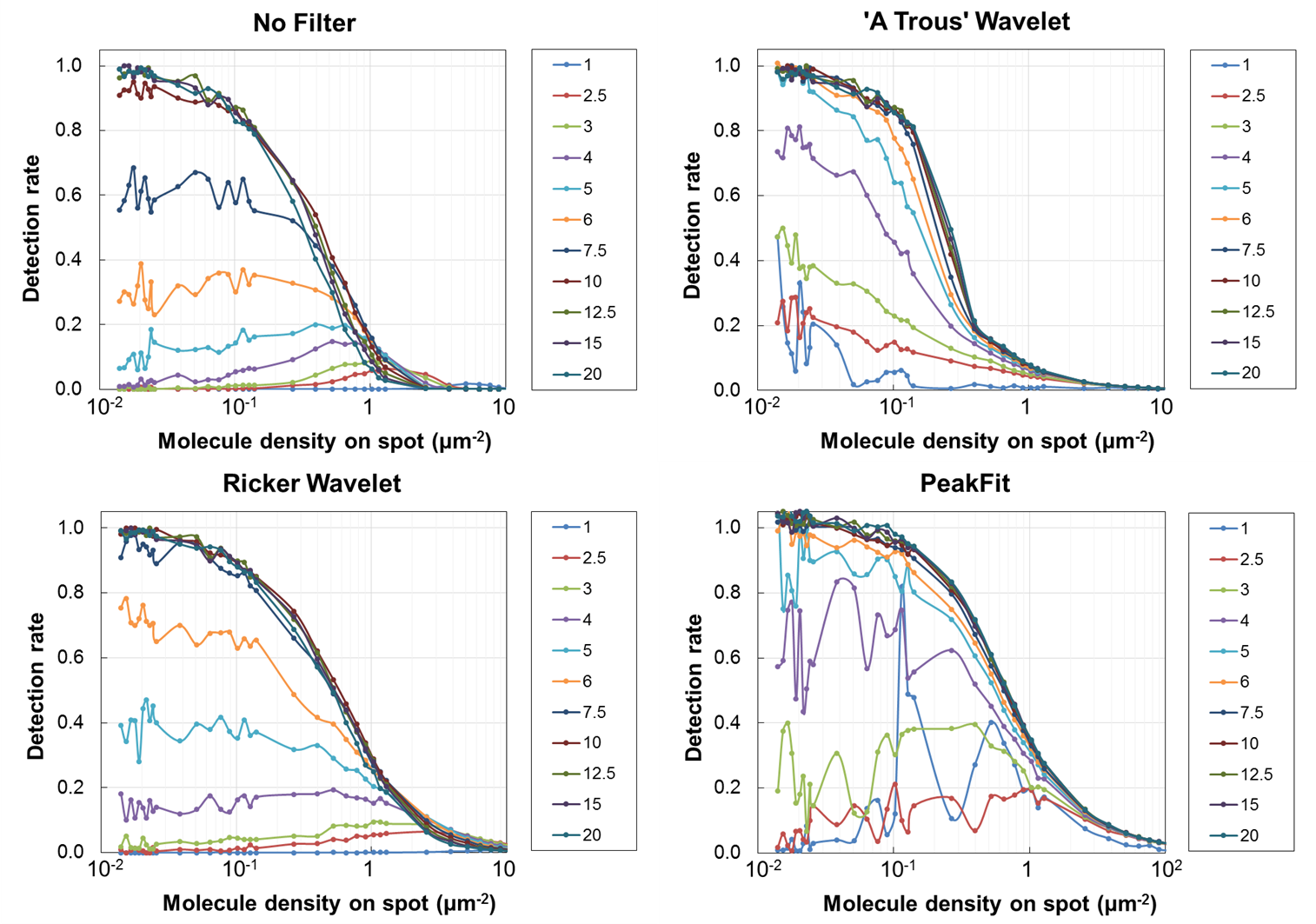 |
| --- |
| **Figure S2:** Image analysis of datasets using methods suited for non-congested arrays. The synthetic image datasets were processed and the accuracy of each single molecule image analysis method was assessed. Datasets are formed of images containing a pre-defined number of single molecules. Each dataset was characterised by a signal to noise ratio (SNR) of the single molecules; SNR values varied between 1 and 20. Accuracy data for each image analysis method is shown. Accuracy improves with increasing SNR. As the number of single molecules per images increases, so does their density on spot, such that the degree of overlap becomes significant and accuracy drops as a result. The figure labels ‘No Filter’, ‘A Trous Wavelet’ and ‘Ricker Wavelet’ represent the results of the intensity thresholding algorithms with either no filters or pre-processing, or pre-processing with the ‘à trous’ or Ricker wavelet transforms, respectively. Similarly, the ‘PeakFit’ figure label represents the results of the peak fitting algorithm. |

| 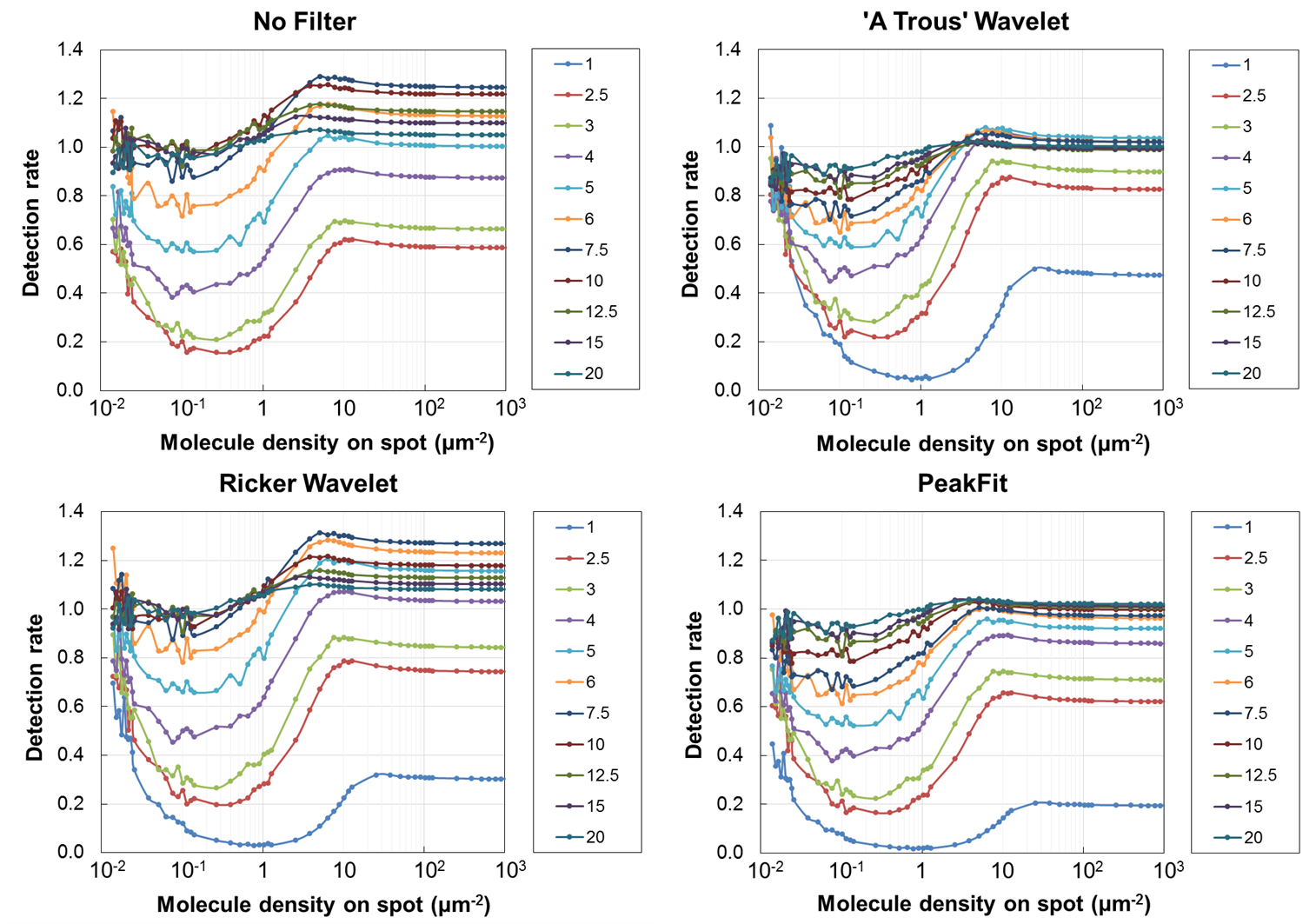 |
| --- |
| **Figure S3:** Image analysis of datasets using methods suited for congested arrays. The density of single molecules as the umber per image sufficiently increases results in a significant degree of overlap i.e. single molecules become difficult to individually distinguish and cannot be accurately enumerated using methods suited for peak detection. The number of single molecules is then estimated from dividing the total intensity by the average single molecule intensity. The same synthetic datasets that were analysed in Figure 2 are now re-analysed. Results show the performance of the algorithms to data with a signal molecule SNR ranging from 1 to 20. The single molecule intensity is determined from non-congested images. The figure labels ‘No Filter’, ‘A Trous Wavelet’ and ‘Ricker Wavelet’ represent the results when using estimates of average single molecule intensity determined using the intensity thresholding algorithms with either no filters or pre-processing, or pre-processing with the ‘à trous’ or Ricker wavelet transforms, respectively. Similarly, the ‘PeakFit’ figure label represents the results when using an estimate of average single molecule intensity determined using the peak fitting algorithm. |

| 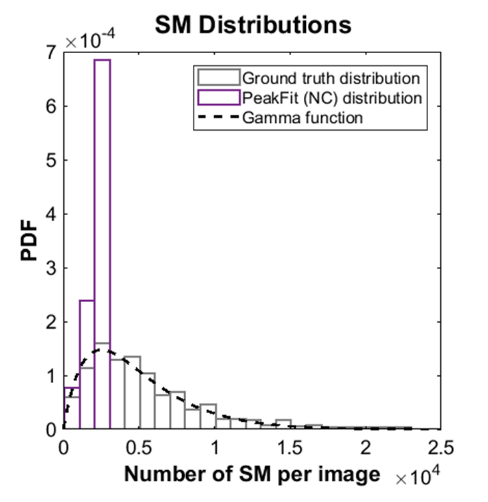 |
| --- |
| **Figure S4:** Synthetic data is generated to model single cell distributions whereby the number of single molecules in the microarray spot follows an asymmetric gamma distribution peaked in the semi-congested regime (k = 2.0, θ = 2.5 × 10^3^). The ground truth distribution and image datasets are generated using the continuous gamma function (dashed black line; k = 2.0, θ = 2.5 × 10^3^). The probability distribution function of histograms comparing the ground truth distribution (grey edges) to the estimated single molecule counts using the peak fitting algorithm (purple edges). This exemplifies the challenge in analysing semi-congested data where neither single molecule or congested image analysis methods are best suited. |
